# Case of Hydrocephalus; Puncture; Death

**Published:** 1861-04

**Authors:** 


### CASE OF HYDROCEPHALUS; PUNCTURE; DEATH.

A girl aged fifteen months was admitted into St. Bartholomew's Hospital, under care of Mr. Paget, August 31, for hydrocephalus. She was a badly nourished child, emaciated and feeble. She was said not to have been born with a large head, but that it commenced to increase two months after birth. It was also ascertained that her parents were first cousins, that her mother was paraplegic, and her father phthisical. Four months before her admission her head was found to measure twenty-seven inches round, but on admission the measurement was only twenty-six. The orbital plate of the frontal bone was much pushed forwards, and hence more nearly in the perpendicular. The eyeballs were displaced inwards and forwards. The bones of the skull were at the interior fontanelle widely apart, the fissure extending for some distance betwixt the frontal and parietal bones. She was quiet in the day, seemed pretty comfortable, but cried most of the night. The thumbs were constantly drawn in on the palms of the hands. She still showed some signs of intelligence, and knew the nurse and sisters. Her appetite was voracious. On September 1st Mr. Paget introduced a capillary trocar by the side of the anterior fontanelle, and allowed six ounces of fluid to escape. Except the trifling pain of the introduction of the instrument, no particular uneasiness was produced by the presence of the trocar. On the 8th, sixteen ounces of fluid were pumped out by means of a small stomach-pump with a gutta percha tube attached. After each of these operations the child was sick for about two hours, but next day it took food, and seemed as well as usual. On the 15th, Mr. Paget drew off eight ounces of fluid, and injected one ounce of an aqueous solution of iodine and iodide of potassium (ten grains of iodine, one scruple of iodide of potassium, and an ounce of water). The operation was borne well, and no unusual effects were produced. No iodism. On the 18th (three days after the injection of the solution), the head measured twenty-seven inches. After each of the first two operations the head collapsed slightly, the space in the anterior fontanelle becoming depressed, and the skin being looser than before. Next day, however, it had regained its previous size and appearance, the fontanelle being as tense as ever. After the third injection, however, the head did not regain its previous dimensions for ten days. On September 20th a fourth operation was performed. Eighteen ounces of fluid were drawn off, and three ounces of the solution injected. The fluid drawn off was rather more colored than usual, being somewhat like that of hydrocele. No iodine or iodide of potassium was detected in it. We may mention that Professor Langenbeck, of Berlin, was present at the operation. He suggested that the trocar should be introduced through the orbital plate of the frontal bone, in order to avoid puncturing the corpus callosum. Mr. Paget, however, preferred to puncture by the side of the anterior fontanelle, as he did in the three previous operations. The operation was performed at about half-past two o'clock in the afternoon. At five o'clock the child became convulsed, and continued so until six or seven o'clock, when the spasm became more permanent, and the child seemed quite rigid. The next morning the eyes were quite bloodshot, and the eyeballs more prominent and drawn in. During the whole of the night the child's limbs remained quite rigid. She did not cry, but she had no sleep. She was very sick. The thumbs, as previously, were spasmodically contracted on the palm, and both the great toes were also spasmodically flexed. The next day (22d) the jaws were quite stiff, the eyeballs sunken. There was less stiffness about the extremities. With the exception of a little milk, she had taken no food since the operation. She died at two o'clock, being just three days after the operation.

*Autopsy.*—Sept. 24—An incision was made into the more depending portion of the head, and about four pints of turbid serum were let out. The brain, which was not very adherent to the parts around, was very soft, and at the back was softened to a pulp. The cerebellum was moderately firm, and was pushed towards the right side. The lining membrane of the general ventricular cavity (the septum lucidum being broken down) was found exceedingly vascular, quite scarlet, the vessels presenting almost a varicose appearance. At the anterior part of the ventricular cavity were deposits of flabby puriform lymph, the fluid being very turbid. The fourth ventricle was carefully examined, and was found to be quite blocked up. The lungs were studded with tubercle, as was also the costal layer of the pleura. The bronchial glands were tuberculous. The liver and spleen were also the seat of tuberculous deposits. The fluid drawn off was examined for iodine and iodide of potassium; the latter only was detected. —*Med. Times and Gazette, Dec.* 22, 1860.

NOTE.—This is the third case of hydrocephalus treated by injections of iodine of which we have knowledge. The first was that of Dr. Brainard, in which the treatment was continued during seven months. At first the effect was favorable, but eventually the disease resumed its course. The second was reported by Dr. Tournesko, of Bucharest. The disease was reported cured after one injection.

The case of Dr. Paget is interesting, for notwithstanding the result was not favorable, the injection of ten grains of iodine and twenty grains of iodide of potassium, at three different times, with no more effect than had been produced by simple tapping, is calculated to encourage further trials. It corresponds to what we have witnessed, for in the first case above alluded to no effects of a serious character were produced by the injections. Fatal symptoms, like those of Dr. Paget's case, came on while no treatment was made use of.

